# Families of Strictly Pseudoconvex Domains and Peak Functions

**DOI:** 10.1007/s12220-017-9912-2

**Published:** 2017-09-06

**Authors:** Arkadiusz Lewandowski

**Affiliations:** Faculty of Mathematics and Computer Science, Institute of Mathematics, Jagiellonian University, Łojasiewicza 6, 30-348 Kraków, Poland

**Keywords:** Strictly pseudoconvex domains, Peak functions, Peak points, Primary 32T40, Secondary 32T15

## Abstract

We prove that given a family $(G_t)$ of strictly pseudoconvex domains varying in $\mathcal {C}^2$ topology on domains, there exists a continuously varying family of peak functions $h_{t,\zeta }$ for all $G_t$ at every $\zeta \in \partial G_t$.

## Introduction

Let $D\subset \mathbb {C}^n$ be a bounded domain and let $\zeta$ be a boundary point of *D*. It is called a *peak point* with respect to $\mathcal {O}(\overline{D})$, the family of functions which are holomorphic in a neighborhood of $\overline{D},$ if there exists a function $f\in \mathcal {O}(\overline{D})$ such that $f(\zeta )=1$ and $f(\overline{D}{\setminus }\{\zeta \})\subset \mathbb {D}:=\{z\in \mathbb {C}:|z|<1\}.$ Such a function is a *peak function for* *D* *at* $\zeta$. The concept of peak functions appears to be a powerful tool in complex analysis with many applications. It has been used to show the existence of (complete) proper holomorphic embeddings of strictly pseudoconvex domains into the unit ball $\mathbb {B}^N$ with large *N* [3, 5], to estimate the boundary behavior of Carathéodory and Kobayashi metrics [1, 7], or to construct the solution operators for $\overline{\partial }$ problem with $L^{\infty }$ or Hölder estimates [4, 10], just to name a few of those applications.

It is well known that every boundary point of strictly pseudoconvex domain is a peak point. Even more is true, in [7] it is showed that, given a strictly pseudoconvex domain *G*, there exists an open neighborhood $\widehat{G}$ of *G*, and a continuous function $h:\widehat{G}\times \partial G\rightarrow \mathbb {C}$ such that for $\zeta \in \partial G$, the function $h(\cdot ;\zeta )$ is a peak function for *G* at $\zeta$.

In a recent paper [2], the following question has been posed:

### Problem 1.1

Let $\rho :\mathbb {D}\times \mathbb {C}^n\rightarrow \mathbb {R}$ be a plurisubharmonic function of class $\mathcal {C}^{2+k}, k\in \mathbb {N}\cup \{0\},$ such that for any $z\in \mathbb {D}$ the truncated function $\rho |_{\{z\}\times \mathbb {C}^n}$ is strictly plurisubharmonic. Define $G_z:=\{w\in \mathbb {C}^n:\rho (z,w)<0\},z\in \mathbb {D}.$ This can be understood as a family of strictly pseudoconvex domains over $\mathbb {D}.$ Does there exist a $\mathcal {C}^k$-continuously varying family $(h_{z,\zeta })_{z\in \mathbb {D},\zeta \in \partial G_z}$ of peak functions for $G_z$ at $\zeta$?

We answer this question affirmatively in the case $k=0$ and under additional assumption that, roughly speaking, the function $\rho$ keeps its regularity up to the set $\Omega \times \mathbb {C}^n$, where $\Omega$ is some open neighborhood of $\overline{\mathbb {D}}$ (however, see Remark 1.5 below). Namely, let us consider the following:

### Situation 1.2

Let $(G_t)_{t\in T}$ be a family of bounded strictly pseudoconvex domains, where *T* is a compact metric space with associated metric *d*. Suppose we have a domain $U\subset \subset \mathbb {C}^n$ such that

1. $$\displaystyle {\bigcup _{t\in T}\partial G_t\subset \subset U},$$
2. for each $t\in T$ there exists a defining function $r_t$ for $G_t$ satisfying with neighborhood $\partial G_t\subset U$ all the conditions (A)–(D) below (see Sect. 2),
3. for any $\varepsilon >0$ there exists a $\delta >0$ such that for any $s,t\in T$ with $d(s,t)\le \delta$ there is $\Vert r_t-r_s\Vert _{\mathcal {C}^2(U)}<\varepsilon$.

Observe that the above setting is completely in the spirit of the formulation of Problem 1.1:

(i) The assumption that all the functions $r_t$ satisfy (A)–(D) with common neighborhood $\partial G_t\subset U$ stays in relation with the fact that in Problem 1.1 all the defining functions for domains $D_z$ have the same domain of definition ($\mathbb {C}^n$).
(ii) The assumption (3) comes from the fact that the function $\rho$ in Problem 1.1 is of class at least $\mathcal {C}^2.$
(iii) The compactness of the set of parameters (*T*) reflects the above-mentioned assumption that $\rho$ continues to be of class $\mathcal {C}^2$ up to $\Omega \times \mathbb {C}^n$, with $\Omega$ being some neighborhood of $\overline{\mathbb {D}}.$

We shall prove the following:

### Theorem 1.3

Let $(G_t)_{t\in T}$ be a family of strictly pseudoconvex domains as in Situation 1.2. Then there exists an $\varepsilon >0$ such that for any $\eta _1<\varepsilon$ there exist an $\eta _2>0$ and positive constants $d_1,d_2$ such that for any $t\in T$ there exist a domain $\widehat{G_t}$ containing $\overline{G_t}$, and functions $h_t(\cdot ;\zeta )\in \mathcal {O}(\widehat{G_t}),\zeta \in \partial G_t$ fulfilling the following conditions:

- $h_t(\zeta ;\zeta )=1, |h_t(\cdot ;\zeta )|<1$ on $\overline{G_t}{\setminus }\{\zeta \}$ (in particular, $h_t(\cdot ;\zeta )$ is a peak function for $G_t$ at $\zeta$),
- $$|1-h_t(z;\zeta )|\le d_1\Vert z-\zeta \Vert , z \in \widehat{G_t}\cap \mathbb {B}(\zeta ,\eta _2),$$
- $$|h_t(z;\zeta )|\le d_2<1, z\in \overline{G_t},\Vert z-\zeta \Vert \ge \eta _1.$$

Moreover, the constants $\varepsilon , \eta _2, d_1, d_2$, domains $\widehat{G_t},$ and functions $h_t(\cdot ;\zeta )$ may be chosen in such a way that for any $\alpha >0$ and any fixed triple $(t_0,\zeta _0,z_0)$, where $t_0\in T, \zeta _0\in \partial G_{t_0},$ and $z_0\in \widehat{G_{t_0}}$, there exists a $\delta >0$ such that whenever the triple $(s, \xi , w)$ satisfies $s\in T, \xi \in \partial G_s, w\in \widehat{G_s},$ and $\max \{d(s,t_0),\Vert \xi -\zeta _0\Vert ,\Vert w-z_0\Vert \}<\delta$, then $|h_{t_0}(z_0;\zeta _0)-h_s(w;\xi )|<\alpha$.

The latter property will be referred to as *continuity*.

### Remark 1.4

It is known that for each $t\in T$ there exists an $\varepsilon =\varepsilon (t)>0$ such that for any $\eta _1<\varepsilon$ there exist a positive $\eta _2=\eta _2(t)<\eta _1$, constants $d_1=d_1(t), d_2=d_2(t)\in \mathbb {R},$ domain $\widetilde{G_t}$ containing $\overline{G_t}$, and functions $h_t(\cdot ;\zeta )\in \mathcal {O}(\widehat{G_t}),\zeta \in \partial G_t$ satisfying (a)–(c). This is a subject of Theorem 19.1.2 from [8]. The strength of our result dwells in the fact that all the constants $\varepsilon , \eta _2, d_1, d_2$ are chosen independently of *t* and in the continuity property.

### Remark 1.5

As noticed by the referee, our result can be strengthened in the spirit of Theorem 5.1 from [6]. It gives the construction of Henkin–Ramírez functions for variable strictly pseudoconvex open sets (with boundaries of class $\mathcal {C}^{2+a,j}$; see Definition 2.5 therein) depending $\mathcal {C}^{1+a,j}$-smoothly on a parameter. Under similar assumptions as in [6], and by merging the method of proof of our Theorem 1.3 with the method of proof of Theorem 5.1 from [6], we can get similar regularity for the dependence of our peak functions on the parameter.

In Sect. 2, we recall some preliminaries concerning the strictly pseudoconvex domains. The proof of Theorem 1.3 is presented in Sect. 3.

## Strictly Pseudoconvex Domains

Let $D\subset \subset \mathbb {C}^n$ be a domain. It is called a *strictly pseudoconvex* if there exist a neighborhood *U* of $\partial D$ and a *defining function* $r:U\rightarrow \mathbb {R}$ of class $\mathcal {C}^2$ and such that

(A) $$D\cap U=\{z\in U:r(z)<0\}$$,
(B) $$(\mathbb {C}^n{\setminus }\overline{D})\cap U=\{z\in U:r(z)>0\},$$
(C) $$\nabla r(z)\ne 0$$ for $$z\in \partial D,$$ where $$\nabla r(z):=\left( \frac{\partial r}{\partial \overline{z}_1}(z),\ldots ,\frac{\partial r}{\partial \overline{z}_n}(z)\right) $$,

together with

$$\begin{aligned} \mathcal {L}_r(z;X)>0\, \text {for}\, z\in \partial D\, \text {and}\, \text {nonzero} X\in T_z^{\mathbb {C}}(\partial D), \end{aligned}$$

where $\mathcal {L}_r$ denotes the Levi form of *r* and $T_z^{\mathbb {C}}(\partial D)$ is the complex tangent space to $\partial D$ at *z*.

It is known that *U* and *r* can be chosen to satisfy (A)-(C) and, additionally,

(D) $\mathcal {L}_r(z;X)>0$ for $z\in U$ and all nonzero $X\in \mathbb {C}^n,$

cf. [9].

Note that for a function *r* as above and a point $\zeta \in \partial G$, Taylor expansion of *r* at $\zeta$ has the following form:

2.1 $$\begin{aligned} r(z)=r(\zeta )-2\text {Re}P(z;\zeta )+\mathcal {L}_{r}(\zeta ;z-\zeta )+o(\Vert z-\zeta \Vert ^2), \end{aligned}$$

where

$$\begin{aligned} \displaystyle { P(z;\zeta ):=-\sum _{j=1}^n\frac{\partial r}{\partial z_j}(\zeta )(z_j-\zeta _j)-\frac{1}{2}\sum _{i,j=1}^n\frac{\partial ^2r}{\partial z_i\partial z_j}(\zeta )(z_i-\zeta _i)(z_j-\zeta _j) } \end{aligned}$$

is the *Levi polynomial of* *r* *at* $\zeta$.

## Proof of Theorem 1.3

We divide the proof into two parts. First we give the construction of $\widehat{G_t}$ and $h_t(\cdot ;\zeta ),t\in T$, and define the constants $\varepsilon , \eta _2, d_1$, and $d_2$, all independent of *t*. This is refinement of the construction from the proof of Theorem 19.1.2 from [8]. Note that in order to get the independence of all the constants from *t*, we must be more careful here. In the second part, we prove the continuity property.

*Construction of* $\widehat{G_t}$ *and* $h_t(\cdot ;\zeta )$ *and the choice of* $\varepsilon , \eta _2, d_1$, *and* $d_2$. *For* $t\in T$ and $\zeta \in \partial G_t$ let $P_t(z;\zeta )$ be the Levi polynomial of $r_t$ at $\zeta$. $\square$

Fix an $\varepsilon _1>0$ such that $\displaystyle {U':=\bigcup \nolimits _{t\in T,\zeta \in \partial G_t}\mathbb {B}(\zeta ,\varepsilon _1)}\subset \subset U.$

There exists a constant $C_1=C_1(t)<1$ such that

$$\begin{aligned} \mathcal {L}_{r_t}(z;X)\ge C_1\Vert X\Vert ^2, \quad z\in U',X\in \mathbb {C}^n. \end{aligned}$$

Indeed, $\mathcal {L}_{r_t}$ is continuous and positive on $U\times (\mathbb {C}^n{\setminus }\{0\})$, so it attains its minimum $C_1(t)>0$ on $\overline{U'}\times \mathbb {S}^{n-1}.$ Since for any nonzero $X\in \mathbb {C}^n$ we have $\frac{X}{\Vert X\Vert }\in \mathbb {S}^{n-1},$ we get the required inequality. Moreover, from the assumption (3) it follows that for *s* from some neighborhood of *t*, we have

$$\begin{aligned} \mathcal {L}_{r_s}(z;X)\ge \frac{C_1(t)}{2}\Vert X\Vert ^2, \quad z\in U',X\in \mathbb {C}^n. \end{aligned}$$

The compactness argument then gives that $C_1$ may be chosen independently of *t*.

Taylor formula (2.1) yields that with some $0<C_2<C_1$ there is

3.1 $$\begin{aligned} r_t(z)\ge -2\text {Re}P_t(z;\zeta )+C_2\Vert z-\zeta \Vert ^2 \end{aligned}$$

for $\Vert z-\zeta \Vert<\varepsilon _2(t)<\varepsilon _1,\zeta \in \partial G_t$, where $\varepsilon _2(t)$ is independent of $\zeta \in \partial G_t$ (and even of $\zeta \in W\subset \subset U,$ some neighborhood of $\partial G_t$—see [11], Proposition II.2.16). Moreover, from the proof of Theorem V.3.6 from [11], it follows that for *s* close enough to *t* we have

$$\begin{aligned} r_s(z)\ge r_s(\zeta )-2\text {Re}P_s(z;\zeta )+\frac{C_2}{2}\Vert z-\zeta \Vert ^2,\quad \zeta \in W,\Vert z-\zeta \Vert <\varepsilon _2(t). \end{aligned}$$

Therefore, for *s* near to *t*, and for $\xi \in \partial G_s$, the following estimate holds true:

$$\begin{aligned} r_s(z)\ge - 2\text {Re}P_s(z;\xi )+\frac{C_2}{2}\Vert z-\xi \Vert ^2,\quad \Vert z-\xi \Vert <\varepsilon _2(t). \end{aligned}$$

The compactness argument then implies that $C_2$ and $\varepsilon _2$ in (3.1) may be chosen independently of *t*.

Let $0<\eta _1<\varepsilon _2$ and $\widehat{\chi }\in \mathcal {C}^{\infty }(\mathbb {R},[0,1])$ be such that $\widehat{\chi }(t)=1$ for $t\le \frac{\eta _1}{2}$ and $\widehat{\chi }(t)=0$ for $t\ge \eta _1.$ Put $\chi (z;\zeta ):=\widehat{\chi }(\Vert z-\zeta \Vert ).$ This is a smooth function on $\mathbb {C}^n\times \mathbb {C}^n$, taking its values in [0, 1].

Define

$$\begin{aligned} \varphi _t(z;\zeta ):=\chi (z;\zeta )P_t(z;\zeta )+(1-\chi (z;\zeta ))\Vert z-\zeta \Vert ^2,\quad z\in \mathbb {C}^n. \end{aligned}$$

Observe that if $\Vert z-\zeta \Vert \le \frac{\eta _1}{2},$ then $\varphi _t(z;\zeta )=P_t(z;\zeta ).$ In particular $\varphi _t(\cdot ;\zeta )\in \mathcal {O}(\mathbb {B}(\zeta ,\frac{\eta _1}{2}))$. Furthermore, for *z* satisfying $\Vert z-\zeta \Vert \ge \frac{\eta _1}{2}$ and $r_t(z)< C_2\frac{\eta _1^2}{8}$ the following estimate holds true:

3.2 $$\begin{aligned} 2\text {Re}\varphi _t(z;\zeta )\ge C_2\frac{\eta _1^2}{8}>0. \end{aligned}$$

Take $0<\eta _t<C_2\frac{\eta _1^2}{8}$ such that the connected component $\widetilde{G_t}$ containing $\overline{G_t}$ of the open set

$$\begin{aligned} G_t\cup \{z\in U':r_t(z)<\eta _t\} \end{aligned}$$

is a strictly pseudoconvex domain, relatively compact in $G_t\cup U'.$ Because of the assumption (3), there exists a positive number $\beta$ such that for *s* close to *t* the connected component $\widetilde{G_s}$ containing $\overline{G_s}$ of the set

$$\begin{aligned} G_s\cup \{z\in U':r_s(z)<\eta _t-\beta \} \end{aligned}$$

is a strictly pseudoconvex domain, relatively compact in $G_s\cup U'.$ Making again use of the compactness of *T*, we conclude that in fact $\eta =\eta _t$ may be taken independently of *t*. Note that, for the family $(\widetilde{G_t})_{t\in T}$, the assumption (3) remains true.

The function $\varphi _t(\cdot ;\zeta )\in \mathcal {C}^{\infty }(\mathbb {C}^n)$ does not vanish on $\widetilde{G_t}{\setminus }\mathbb {B}(\zeta ,\frac{\eta _1}{2})$ and is in $\mathcal {O}(\mathbb {B}(\zeta ,\frac{\eta _1}{2}))$. Therefore $\bar{\partial }\frac{1}{\varphi _t(\cdot ;\zeta )}$ defines a $\bar{\partial }$-closed $\mathcal {C}^{\infty }$ form

$$\begin{aligned} \displaystyle {\alpha _t(\cdot ;\zeta )=\sum _{j=1}^n\alpha _{t,j}(\cdot ;\zeta )d\bar{z}_j} \end{aligned}$$

on $$\widetilde{G_t},$$ where

$$\begin{aligned} \displaystyle { \alpha _{t,j}={\left\{ \begin{array}{ll} 0,&{}z\in \widetilde{G_t}\cap \mathbb {B}(\zeta ;\frac{\eta _1}{2}),\\ -\frac{\partial \varphi _t}{\partial \bar{z}_j}(z;\zeta )\cdot \frac{1}{\varphi _t^2(z;\zeta )},&{}z\in \widetilde{G_t} {\setminus }\mathbb {B}(\zeta ;\frac{\eta _1}{2}). \end{array}\right. }} \end{aligned}$$

Thanks to (3.2) we have $\Vert \alpha _{t,j}(\cdot ;\zeta )\Vert _{\widetilde{G_t}}\le C_3$, where, utilizing the compactness of *T* together with the assumption (3), we deliver that $C_3$ is independent of *t* and $\zeta \in \partial G_t$. [11, Theorem V.2.7] gives then the functions $v_t(\cdot ;\zeta )\in \mathcal {C}^{\infty }(\widetilde{G_t})$ with $\bar{\partial }v_t(\cdot ;\zeta )=\alpha _t(\cdot ;\zeta )$ and

$$\begin{aligned} \Vert v_t(\cdot ;\zeta )\Vert _{\widetilde{G_t}}\le C_4, \end{aligned}$$

where $C_4$ does not depend on $\zeta \in \partial G_t.$ Moreover, by [11, Theorem V.3.6] and the compactness of *T*, $C_4$ may be chosen to be independent of *t*.

Define

$$\begin{aligned} f_t(\cdot ;\zeta ):=\frac{1}{\varphi _t(\cdot ;\zeta )}+C_4-v_t(\cdot ;\zeta ),\quad z\in \widetilde{G_t}{\setminus } Z_t(\zeta ), \end{aligned}$$

where

$$\begin{aligned} Z_t(\zeta ):=\{z\in \widetilde{G_t}:\varphi _t(z;\zeta )=0\}. \end{aligned}$$

Then $f_t(\cdot ;\zeta )\in \mathcal {O}(\widetilde{G_t}{\setminus } Z_t(\zeta ))$ as well as

$$\begin{aligned} \text {Re}f_t(\cdot ;\zeta )>0 \end{aligned}$$

on the set $(\widetilde{G_t}{\setminus }\mathbb {B}(\zeta ,\frac{\eta _1}{2}))\cup (\overline{G_t}{\setminus }\{\zeta \})$, in virtue of (3.1) and (3.2). Since for any $\zeta \ne z_0\in \partial {G_t}\cap \overline{\mathbb {B}}(\zeta ,\frac{\eta _1}{2})$ there exists a neighborhood $U_{z_0}$ of $z_0$ such that $\text {Re}f_t(\cdot ;\zeta )>0$ on $U_{z_0}$, we conclude that there exists a neighborhood $U_{t,\zeta }$ of $\overline{G_t}{\setminus }\{\zeta \}$ such that the function

$$\begin{aligned} h_t(\cdot ;\zeta ):=\text {exp}(-g_t(\cdot ;\zeta )), \end{aligned}$$

where $g_t(\cdot ;\zeta ):=\frac{1}{f_t(\cdot ;\zeta )}$, is holomorphic on $H_{t,\zeta }:=(\widetilde{G_t}{\setminus }\mathbb {B}(\zeta ,\frac{\eta _1}{2}))\cup U_{t,\zeta }.$ Note that $h_t$ takes its values in $\mathbb {D}.$

There exists a $C_5>0,$ independent of *t*, such that

$$\begin{aligned} |P_t(z;\zeta )|\le C_5\Vert z-\zeta \Vert ,\quad \zeta , z\in U'. \end{aligned}$$

Therefore, since for $0<\eta _2<\min \big \{\frac{\eta _1}{2},\frac{1}{4C_4C_5}\big \}$, which now is independent of *t*, and for $z\in (\widetilde{G_t}\cap \mathbb {B}(\zeta ,\eta _2)){\setminus } Z_t(\zeta )$ the following equality holds true:

$$\begin{aligned} g_t(z;\zeta )=\frac{P_t(z;\zeta )}{1-P_t(z;\zeta )(v_t(z;\zeta )-C_4)}, \end{aligned}$$

we conclude that $g_t(\cdot ;\zeta )$ is bounded near $Z_t(\zeta )$, which yields it extends to be holomorphic on $\widetilde{H_{t,\zeta }}:=H_{t,\zeta }\cup (\mathbb {B}(\zeta ,\eta _2)\cap \widetilde{G_t}).$

Now $\widetilde{H_{t,\zeta }}$ depends on $\zeta$, but using the inclusion $\overline{G_t}\subset \widetilde{H_{t,\zeta }}$, we may find some $\widehat{G_t},$ strictly pseudoconvex domain which is independent on $\zeta \in \partial G_t$, such that $\overline{G_t}\subset \widehat{G_t}\subset \widetilde{H_{t,\zeta }}$ for each $\zeta \in \partial G_t$, and with the property that $h_t(\cdot ;\zeta )\in \mathcal {O}(\widehat{G_t}), \zeta \in \partial G_t$ (use the joint continuity of $\varphi _t$ with respect to *z* and $\zeta$ to shrink $\widetilde{H_{t,\zeta }}$ little bit to get some domain with desired properties, independent on $\xi$ close to $\zeta$, and finally apply the compactness of $\partial G_t$).

Let $C_6$, independent on *t* and $\zeta \in \partial G_t$, such that for $z\in \widehat{G_t}$ with $\Vert z-\zeta \Vert <\eta _2$ we have

$$\begin{aligned} |g_t(z;\zeta )|\le \frac{C_5\Vert z-\zeta \Vert }{1-2C_4C_5\Vert z-\zeta \Vert }\le C_6\Vert z-\zeta \Vert . \end{aligned}$$

This implies

3.3 $$\begin{aligned} |1-h_t(z;\zeta )|\le C_7|g_t(z;\zeta )|\le C_6 C_7\Vert z-\zeta \Vert =:d_1\Vert z-\zeta \Vert \end{aligned}$$

for $z\in \widehat{G_t},\Vert z-\zeta \Vert <\eta _2,\zeta \in \partial G_t$, if only $C_7$ is chosen so that

$$\begin{aligned} |e^{\lambda }-1|\le C_7|\lambda |,\quad |\lambda |\le C_6\eta _2. \end{aligned}$$

In particular, $d_1$ does not depend on *t* and we have $h_t(\zeta ;\zeta )=1.$

Furthermore, for $z\in \overline{G_t},\Vert z-\zeta \Vert \ge \eta _1$ there is

$$\begin{aligned} \text {Re}g_t(z;\zeta )&=\Vert z-\zeta \Vert ^2\frac{1+\Vert z-\zeta \Vert ^2(C_4-\text {Re}v_t(z;\zeta ))}{|1-\Vert z-\zeta \Vert ^2(v_t(z;\zeta )-C_4)|^2}\\&\ge \frac{\eta _1^2}{(1+2(\text {diam}U)^2C_4)^2}=:C_8, \end{aligned}$$

which gives

$$\begin{aligned} |h_t(z;\zeta )|\le e^{-C_8}=:d_2<1. \end{aligned}$$

Observe that $d_2$ is independent on *t*. $\square$

### Proof of continuity

Fix $\alpha >0, t_0\in T,\zeta _0\in \partial G_{t_0},$ and $z_0\in \widehat{G_{t_0}}$. Let $K_0$ be a compact subset of $\widetilde{G_{t_0}},$ containing in its interior the set $\overline{G_{t_0}}\cup \{z_0\}.$ In the sequel, we shall use the following convention: whenever we say that the triple $(s,\xi ,w)$ is near to $(t_0,\zeta _0,z_0),$ it will carry the additional information that $\xi \in \partial G_s,w\in \widehat{G_s}$, unless explicitly stated otherwise.

Observe that for $(s,\xi )$ close to $(t_0,\zeta _0)$ (even without requiring that $\xi \in \partial G_s$), and any $z\in U'$ we have

$$\begin{aligned} |P_{t_0}(z;\zeta _0)-P_s(z;\xi )|<M_0\alpha \end{aligned}$$

with some positive $M_0.$ In particular, for *w* close to $z_0$ the following estimate is true

$$\begin{aligned} |P_{t_0}(z_0;\zeta _0)-P_s(w;\xi )|<M_1\alpha , \end{aligned}$$

where $$M_1:=M_0+1.$$

Further, using the fact that all the functions $\varphi _t$ are continuous as functions of both variables, we conclude that for $(s,\xi )$ close to $(t_0,\zeta _0)$ we have

$$\begin{aligned} \Vert \varphi _{t_0}(\cdot ;\zeta _0)-\varphi _s(\cdot ;\xi )\Vert _{U'}<M_2\alpha \end{aligned}$$

with some positive $M_2.$

For $(s,\xi )$ near $(t_0,\zeta _0)$ we have

$$\begin{aligned} \left\| \frac{\partial \varphi _{t_0}}{\partial \bar{z}_j}(\cdot ;\zeta _0)-\frac{\partial \varphi _{s}}{\partial \bar{z}_j}(\cdot ;\xi )\right\| _{U'}<M_3\alpha \end{aligned}$$

with some positive $M_3.$ Furthermore, for $(s,\xi )$ close to $(t_0,\zeta _0)$ and $z\in \widetilde{G_s}\cap \widetilde{G_{t_0}}$, the following estimates hold true:

(I) If $$z\notin \mathbb {B}(\zeta _0,\frac{\eta _1}{2})\cup \mathbb {B}(\xi ,\frac{\eta _1}{2})$$, then
  $$\begin{aligned} |\alpha _{t_0,j}(z;\zeta _0)-\alpha _{s,j}(z;\xi )|< L\alpha , \end{aligned}$$
  where positive constant *L* does not depend on *z* as above. Indeed,
  $$\begin{aligned}&\left| \frac{\frac{\partial \varphi _{t_0}}{\partial \bar{z}_j}(z;\zeta _0)}{\varphi ^2_{t_0}(z;\zeta _0)}-\frac{\frac{\partial \varphi _{s}}{\partial \bar{z}_j}(z;\xi )}{\varphi ^2_{s}(z;\xi )}\right| = \left| \frac{\varphi _s^2(z;\xi )\frac{\partial \varphi _{t_0}}{\partial \bar{z}_j}(z;\zeta _0)-\varphi _{t_0}^2(z;\zeta _0)\frac{\partial \varphi _{s}}{\partial \bar{z}_j}(z;\xi )}{\varphi ^2_{t_0}(z;\zeta _0)\varphi _s^2(z;\xi )}\right| \\&\quad \le \frac{64}{C_2^2\eta _1^4}\left| \varphi _s^2(z;\xi )\frac{\partial \varphi _{t_0}}{\partial \bar{z}_j}(z;\zeta _0)-\varphi _{t_0}^2(z;\zeta _0)\frac{\partial \varphi _{s}}{\partial \bar{z}_j}(z;\xi )\right| \\&\quad \le \frac{64}{C_2^2\eta _1^4}\left( \Vert \varphi ^2_s\Vert _{U'}\left\| \frac{\partial \varphi _{t_0}}{\partial \bar{z}_j}(\cdot ;\zeta _0){-}\frac{\partial \varphi _{s}}{\partial \bar{z}_j}(\cdot ;\xi )\right\| _{U'}{+}\left\| \frac{\partial \varphi _s}{\partial \bar{z}_j}(\cdot ;\xi )\right\| _{U'}{\left\| {\varphi _{s}^2} - {\varphi _{t_0}^2}\right\| }_{U'}\right) \\&\quad \le \frac{64}{C_2^2\eta _1^4}(L_1M_3\alpha +L_2M_2\alpha )=:L\alpha , \end{aligned}$$
  where the first inequality is the consequence of (3.2).
(II) If $z\in \mathbb {B}(\zeta _0,\frac{\eta _1}{2})\cup \mathbb {B}(\xi ,\frac{\eta _1}{2})$: Observe that letting $\xi$ close to $\zeta _0$, we may make the balls arbitrarily close to each other. Using then the assumption (3), the fact that $\eta$ were chosen to be strictly smaller than $C_2\frac{\eta _1^2}{8}$, and the strictness of uniform estimate (3.2), we see that for $(s,\xi )$ close enough to $(t_0,\zeta _0)$ the estimate similar to the previous one holds true for $\displaystyle {z\in S:=\bigcup \nolimits _{w:\Vert w-\zeta _0\Vert =\frac{\eta _1}{2}}\mathbb {B}(w,\gamma )}$ with some sufficiently small $\gamma >0$ (and is independent on such *z*). Additionally, $(s,\xi )$ may be chosen so that $S':=(\mathbb {B}(\zeta _0,\frac{\eta _1}{2})\cup \mathbb {B}(\xi ,\frac{\eta _1}{2})){\setminus } S\subset \mathbb {B}(\zeta _0,\frac{\eta _1}{2})\cap \mathbb {B}(\xi ,\frac{\eta _1}{2})$.

Noting that for $z\in S'$ and $(s,\xi )$ as above $\alpha _{t_0,j}(z;\zeta _0)=\alpha _{s,j}(z;\xi )=0,$ we conclude that

$$\begin{aligned} \Vert \alpha _{t_0}(\cdot ;\zeta _0)-\alpha _s(\cdot ;\xi )\Vert _{\widetilde{G_{t_0}}\cap \widetilde{G_s}}\le M_4\alpha \end{aligned}$$

with some positive $M_4.$

Ofcourse $\overline{G_{t_0}}\subset \widetilde{G_{t_0}}$. This yields that for *s* close to $t_0$ we have $\overline{G_{t_0}}\subset \widetilde{G_s}$ as well as $\overline{G_s}\subset \widetilde{G_{t_0}}$ (the assumption (3) remains true for the family $(\widetilde{G_t})_{t\in T}$). For *s* close to $t_0$ we may now pick some $G_{t_0,s}$, a strictly pseudoconvex domain with smooth boundary and such that

$$\begin{aligned} \overline{G_s}\cup \overline{G_{t_0}}\subset K_{0}\subset \subset G_{t_0,s}\subset \subset \widetilde{G_s}\cap \widetilde{G_{t_0}}. \end{aligned}$$

Again thanks to the property (3), $G_{t_0,s}$ may be chosen independently of *s* if *s* is close enough to $t_0$. For such *s*, denote it by $G^{t_0}.$ Then, using Lemma 2 from [7], we find some positive constant $\Gamma$ such that

$$\begin{aligned} \Vert v_{t_0}(\cdot ;\zeta _0)-v_s(\cdot ;\xi )\Vert _{K_{0}}\le \Gamma \Vert \alpha _{t_0}(\cdot ;\zeta _0)-\alpha _s(\cdot ;\xi )\Vert _{G^{t_0}}\le \Gamma M_4\alpha =:M_5\alpha . \end{aligned}$$

Notice that $\Gamma$ may be chosen independently of *s*. Consequently, for $(s,\xi ,w)$ close to $(t_0,\zeta _0,z_0)$ there is

$$\begin{aligned} |v_{t_0}(z_0;\zeta _0)-v_s(w;\xi )|\le |v_{t_0}(z_0;\zeta _0)-v_{t_0}(w;\zeta _0)|+|v_{t_0}(w;\zeta _0)-v_s(w;\xi )|\le M_6\alpha \end{aligned}$$

for some positive $M_6$ (use the smoothness of $v_{t_0}(\cdot ;\zeta _0)$).

There are two cases to be considered:

Case 1.: $z_0\in H_{t_0,\zeta _0}\cap \text {int}K_{0}$. Then $\varphi _{t_0}(z_0;\zeta _0)\ne 0$ and for $(s,\xi ,w)$ near $(t_0,\zeta _0,z_0)$ we have $\varphi _s(w;\xi )\ne 0.$ For such $(s,\xi ,w)$ we have

$$\begin{aligned} |f_{t_0}(z_0;\zeta _0)-f_s(w;\xi )|\le & {} \left| \frac{1}{\varphi _{t_0}(z_0;\zeta _0)}-\frac{1}{\varphi _s(w;\xi )}\right| +|v_{t_0}(z_0;\zeta _0)-v_s(w;\xi )|\\\le & {} \left| \frac{\varphi _s(w;\xi )-\varphi _{t_0}(z_0;\zeta _0)}{\varphi _{t_0}(z_0;\zeta _0)\varphi _s(w;\xi )}\right| +M_6\alpha . \end{aligned}$$

Considering the last but one term, its denominator is bounded below by some positive constant for $(s,\xi ,w)$ close to $(t_0,\zeta _0,z_0)$, and the numerator is estimated from above by $(M_2+1)\alpha .$ Thus for $(s,\xi ,w)$ close to $(t_0,\zeta _0,z_0)$

$$\begin{aligned} |f_{t_0}(z_0;\zeta _0)-f_s(w;\xi )|\le M_7\alpha \end{aligned}$$

for some positive $M_7.$

In our situation, the function $g_{t_0}(\cdot ;\zeta _0)$ is holomorphic in a neighborhood of $z_0$ and so is $g_s(\cdot ;\xi )$ for $(s,\xi )$ close to $(t_0,\zeta _0).$ We conclude that for $(s,\xi ,w)$ close to $(t_0,\zeta _0,z_0)$ there is

$$\begin{aligned} |g_{t_0}(z_0;\zeta _0)-g_s(w;\xi )|\le M_8\alpha \end{aligned}$$

for some positive $M_8$, and

$$\begin{aligned} |h_{t_0}(z_0;\zeta _0)-h_s(w;\xi )|=\big |\text {exp}(-g_{t_0}(z_0;\zeta _0))-\text {exp}(-g_s(w;\xi ))\big |\le M_9\alpha \end{aligned}$$

for some positive $M_9.$

Case 2.: $$z_0\in (\widetilde{G_{t_0}}\cap \mathbb {B}(\zeta _0,\eta _2))\cap \text {int}K_{t_0}.$$

(I) Suppose $\varphi _{t_0}(z_0;\zeta _0)\ne 0.$ It is equivalent to $P_{t_0}(z_0;\zeta _0)\ne 0.$ This yields that $P_s(w;\xi )\ne 0$ for $(s,\xi ,w)$ close to $(t_0,\zeta _0,z_0)$. Then
  $$\begin{aligned}&|g_{t_0}(z_0;\zeta _0)-g_s(w;\xi )|\\&\quad =\left| \frac{P_{t_0}(z_0;\zeta _0)}{1-P_{t_0}(z_0;\zeta _0)(v_{t_0}(z_0;\zeta _0)-C_4)}-\frac{P_{s}(w;\xi )}{1-P_{s}(w;\xi )(v_{s}(w;\xi )-C_4)}\right| \\&\quad \le N|P_{t_0}(z_0;\zeta _0)-P_s(w;\xi )|+N|P_{t_0}(z_0;\zeta _0)P_s(w;\xi )\Vert v_{t_0}(z_0;\zeta _0)-v_s(w;\xi )|\\&\quad \le NM_1\alpha +N'M_6\alpha =:M_{10}\alpha , \end{aligned}$$
  and similarly as in the previous case
  $$\begin{aligned} |h_{t_0}(z_0;\zeta _0)-h_s(w;\xi )|\le M_{11}\alpha \end{aligned}$$
  with some positive $N,N',M_{10}$, and $M_{11}.$
(II) Suppose $\varphi _{t_0}(z_0;\zeta _0)=0.$ This is equivalent to $P_{t_0}(z_0;\zeta _0)=0.$ Then for some positive $\rho$ we have $\mathbb {B}(z_0,\rho )\subset \subset K_0\cap \mathbb {B}(\zeta _0,\eta _2).$ Similarly, for $(s,\xi )$ close to $(t_0,\zeta _0)$ there is $\mathbb {B}(z_0,\rho )\subset \subset K_0\cap \mathbb {B}(\xi ,\eta _2).$ Therefore, because of the choice of $d_1$ in (3.3), for $(s,\xi ,w)$ close to $(t_0,\zeta _0,z_0),w\in \mathbb {B}(z_0,\frac{\rho }{2})$ there is
  $$\begin{aligned} |h_{t_0}(w;\zeta _0)-h_s(w;\xi )|\le & {} |1-h_{t_0}(w;\zeta _0)|+|1-h_s(w;\xi )|\\\le & {} d_1(\Vert w-\zeta _0\Vert +\Vert w-\xi \Vert )\le 2d_1\eta _2. \end{aligned}$$
  Consequently, since the functions $h_{t_0}(\cdot ;\zeta _0)-h_s(\cdot ;\xi )$ are holomorphic in suitable neighborhood of $z_0$ for $(s,\xi )$ close to $(t_0,\zeta _0)$, for some positive $\widetilde{\rho }<\frac{\rho }{2}$, for every $x,y\in \mathbb {B}(z_0,\frac{\widetilde{\rho }}{2})$ we have
  3.4 $$\begin{aligned} |h_{t_0}(x;\zeta _0)-h_s(x;\xi )-h_{t_0}(y;\zeta _0)+h_s(y;\xi )|\le \alpha . \end{aligned}$$
  Moreover, $\widetilde{\rho }$ may be chosen so that for $v,w\in \mathbb {B}(z_0,\frac{\widetilde{\rho }}{2})$ there is
  3.5 $$\begin{aligned} |h_{t_0}(v;\zeta _0)-h_{t_0}(w;\zeta _0)|\le \alpha , \end{aligned}$$
  by continuity of $h_{t_0}(\cdot ;\zeta _0).$ Fix some $w_0\in \mathbb {B}(z_0,\frac{\widetilde{\rho }}{2})$ such that $P_{t_0}(w_0;\zeta _0)\ne 0.$ Then for $(s,\xi )$ near $(t_0,\zeta _0)$, by virtue of the subcase (I), we have
  $$\begin{aligned}|h_{t_0}(w_0;\zeta _0)-h_s(w_0;\xi )|\le \alpha .\end{aligned}$$
  Finally, for $w\in \mathbb {B}(z_0,\frac{\widetilde{\rho }}{2})$ and $(s,\xi )$ close to $(t_0,\zeta _0)$ we have
  $$\begin{aligned} |h_{t_0}(z_0;\zeta _0)-h_s(w;\xi )|\le & {} |h_{t_0}(z_0;\zeta _0)-h_{t_0}(w_0;\zeta _0)|+|h_{t_0}(w_0;\zeta _0)-h_s(w_0;\xi )|\\&+|h_s(w_0;\xi )-h_s(w;\xi )|\le \alpha +\alpha +2\alpha =4\alpha , \end{aligned}$$
  where the last estimate follows from (3.4) and (3.5), which leads us to the conclusion. $\square$

## References

[1] Bedford E, Fornæss JE (1978). Biholomorphic maps of weakly pseudoconvex domains. Duke Math. J..

[2] Deng F, Guan Q, Zhang L (2016). Properties of squeezing functions and global transformations of bounded domains. Trans. Am. Math. Soc..

[3] Drnovšek BD (2015). Complete proper holomorphic embeddings of strictly pseudoconvex domains into balls. J. Math. Anal. Appl..

[4] Fornæss JE (1986). Sup-norm estimates for $$\overline{\partial }$$ in $${\mathbb{C}}^2$$. Ann. Math..

[5] Forstnerič F (1986). Embedding strictly pseudoconvex domains into balls. Trans. Am. Math. Soc..

[6] Gong, X., Kim, K.-T.: The $$\bar{\partial }$$-equation on variable strictly pseudoconvex domains (preprint). arXiv:1505.07042v1

[7] Graham I (1975). Boundary behavior of the Carathéodory and Kobayashi metrics on strongly pseudoconvex domains in $${\mathbb{C}}^n$$ with smooth boundary. Trans. Am. Math. Soc..

[8] Jarnicki M, Pflug P (2014). Invariant Distances and Metrics in Complex Analysis.

[9] Krantz, S.G.: Function Theory of Several Complex Variables, reprint of the 1992 ed. AMS Chelsea Publishing, Providence, RI (2001)

[10] Range RM (1978). The Carathéodory metric and holomorphic maps on a class of weakly pseudoconvex domains. Pacific J. Math..

[11] Range RM (1986). Holomorphic Functions and Integral Representations in Several Complex Variables, Graduate Texts in Mathematics.

